# Phase Unlocking and the Modulation of Tropopause‐Level Trace Gas Advection by the Quasibiennial Oscillation

**DOI:** 10.1029/2021JD036142

**Published:** 2022-11-10

**Authors:** Kasturi Shah, Susan Solomon, Douglas Kinnison, Qiang Fu, David W. J. Thompson

**Affiliations:** ^1^ Department of Earth, Atmospheric and Planetary Sciences Massachusetts Institute of Technology Cambridge MA USA; ^2^ Atmospheric Chemistry Observations and Modeling National Center for Atmospheric Research Boulder CO USA; ^3^ Department of Atmospheric Sciences University of Washington Seattle WA USA; ^4^ Department of Atmospheric Science Colorado State University Fort Collins CO USA

## Abstract

Open questions about the modulation of near‐surface trace gas variability by stratosphere‐troposphere tracer transport complicate efforts to identify anthropogenic sources of gases such as CFC‐11 and N_2_O and disentangle them from dynamical influences. In this study, we explore one model's modulation of lower stratospheric tracer advection by the quasi‐biennial oscillation (QBO) of stratospheric equatorial zonal‐mean zonal winds at 50 hPa. We assess instances of coherent modulation versus disruption through phase unlocking with the seasonal cycle in the model and in observations. We quantify modeled advective contributions to the temporal rate of change of stratospheric CFC‐11 and N_2_O at extratropical and high‐latitudes by calculating a transformed Eulerian mean (TEM) budget across isentropic surfaces from a 10‐member WACCM4 ensemble simulation. We find that positive interannual variability in seasonal tracer advection generally occurs in the easterly QBO phase, as in previous work, and briefly discuss physical mechanisms. Individual simulations of the 10‐member ensemble display phase‐unlocking disruptions from this general pattern due to seasonally varying synchronizations between the model's repeating 28‐month QBO cycle and the 12‐month seasonal cycle. We find that phase locking and unlocking patterns of tracer advection calculations inferred from observations fall within the envelope of the ensemble member results. Our study bolsters evidence for variability in the interannual stratospheric dynamical influence of CFC‐11 near‐surface concentrations by assessing the QBO modulation of lower stratospheric advection via synchronization with the annual cycle. It identifies a likely cause of variations in the QBO influence on tropospheric abundances.

## Key Points

- Synchronizations between modeled QBO and annual cycles of advective contributions to temporal changes of N_2_O and CFC‐11 are identified
- Seasonal unlocking disrupts these synchronizations of the interannual tropopause‐level advection anomalies
- Observed behaviors of seasonal phase locking and unlocking generally fall within the range of model calculations

## 1 Introduction

Trace gas near‐surface variability is modulated by the quasi‐biennial oscillation (QBO) of zonal‐mean zonal winds in the stratosphere (Nevison & Kinnison, 2004; Ray et al., 2020; Ruiz et al., 2021). However, uncertainties in the extent of stratospheric dynamical influence on near‐surface concentrations impede efforts to disentangle the influence of dynamical contributions and anthropogenic sources on measured annual changes in near‐surface tracer concentrations. Recent detection of an unexpected uptick in emissions (and possible illegal production) of the ozone‐depleting substance trichlorofluoromethane (CFC‐11) led to efforts to curb anthropogenic emissions, resulting in a robust and sharp decline in global CFC‐11 emissions in 2019–2020 (Montzka et al., 2018, 2021). With just 40%–60% of unexpected emissions attributed to eastern China 2014–2017 (Rigby et al., 2019) and with a detected decline over 2017–2018 in Chinese CFC‐11 production (Park et al., 2021), it is apparent that CFC‐11 emissions have additional sources such as chlorofluorocarbon banks (Lickley et al., 2020, 2021). Furthermore, these emissions are also subject to stratospheric dynamical influences, particularly modulation by the QBO (Ray et al., 2020; Ruiz et al., 2021). In the stratosphere, an important dynamical influence on stratospheric tracer concentrations over interannual timescales is changing synchronization between the QBO and seasonal cycle (Gray & Dunkerton, 1990; Hamilton, 1989). In this study, we calculate the temporal rate of change of tracer concentrations due to lower stratospheric advection and assess how the QBO and seasonal cycle jointly modulate simulated intrusions of N_2_O and CFC‐11 poor air from the stratosphere into the troposphere. Notably, we present evidence of interannual variations in the interactions between the two in the model.

N_2_O and CFC‐11 are relatively inert in the troposphere and are destroyed in the stratosphere. The primary entry‐point of air from the troposphere into the stratosphere is at the tropical tropopause (Holton et al., 1995). In the stratosphere, a parcel of air is transported by the large‐scale overturning circulation called the Brewer‐Dobson Circulation (BDC). Air upwells at tropical latitudes through much of the stratosphere. In the mid‐to‐upper stratosphere, N_2_O and CFC‐11 are photochemically destroyed as air parcels are lofted upwards and/or laterally. Typically, N_2_O loss occurs higher in the stratosphere than CFC‐11 loss because the former photolyzes at shorter wavelengths (Nevison & Kinnison, 2004). Air starts downwelling at mid‐to‐high latitudes and altitudes, subsiding through the depth of the stratosphere and into the troposphere (Hamilton & Fan, 2000).

On interannual timescales, this stratosphere‐troposphere exchange can be modulated by modes of dynamical variability. The QBO is a quasiperiodic oscillation of the zonal‐mean zonal winds from easterly to westerly in the equatorial stratosphere, with downward descending alternating shear regions and an average period of 28–29 months with a range of about 22–34 months (Pascoe et al., 2005). It is the dominant mode of interannual variability in the stratospheric tropics and subtropics and influences extratropical stratospheric dynamics via its induced secondary mean meridional circulation (Baldwin et al., 2001; Choi et al., 2002; Plumb & Bell, 1982; Dunkerton, 1985; Reed, 1962; Ribera et al., 2004; Wallace, 1967). QBO variations have been detected in observed lower stratospheric tracer concentrations (Schoeberl et al., 2008) as well as in ozone (Gray & Dunkerton, 1990). Changes in tropospheric ozone driven by the stratospheric circulation, QBO and El Niño/Southern Oscillation (ENSO) have been detected in satellite measurements (Neu et al., 2014). A correlation between interannual variability of N_2_O growth rate and ENSO has been detected and attributed to changes in soil and ocean emissions (Ishijima et al., 2009; Nevison et al., 2007; Thompson et al., 2013; Thompson et al., 2019), however, the link between ENSO and the QBO remains unclear (Coy et al., 2017). Signatures of the QBO have also been detected in measured long‐lived surface concentrations of greenhouse gases such as CH_4_ (Hamilton and Fan, 2000) and N_2_O (Hamilton & Fan, 2000; Ruiz et al., 2021).

Previous studies have emphasized that phases of the annual cycle can synchronize (i.e., “lock”) and unsynchronize (i.e., “unlock”) from phases of the QBO (e.g., Gray & Dunkerton, 1990; Hamilton, 1989). Phase locking and unlocking chiefly arise because the periodicity of the QBO and annual cycle are often not divisible by a common denominator and because the QBO's periodicity is variable. Variability in the period of the QBO can also arise due to phase locking patterns between the QBO and tropical upwelling (Rajendran et al., 2015; Yulaeva et al., 1994). Upwelling influences the progression of zonal winds patterns, thus impeding the descent of equatorial winds particularly during winter‐spring seasons when planetary wave activity is high, lengthening the period of the QBO, and allowing for both to be modulated by the annual cycle (Dunkerton, 1991; Saravan, 1990).

The QBO influence on tracer advection varies depending on whether the QBO is phase locked or unlocked to the annual cycle in a given season. Synchronization between the QBO and the annual cycle has a strong influence on tracer transport, although other processes, such as ENSO, can also become important at times. Modulation of ozone transport by the QBO induces subtropical ozone anomalies maximizing in the winter‐spring season in both hemispheres (Gray & Dunkerton, 1990). Lateral seasonal transport of ozone from the mid‐latitudes into the tropics is strongly modulated by the QBO (Hamilton K., 1989). The QBO not only modulates stratospheric upwelling, but also modulates the stratospheric loss of gases, such as N_2_O and CFC‐11, and downwelling of this tracer‐depleted air to the surface (Ruiz D. J. et al., 2021). Imprints of the QBO modulation are detectable in the surface variability of these gases. Chemical transport models (Ruiz et al., 2021), observations (Nevison & Kinnison, 2004), and general circulation model simulations (Nevison & Kinnison, 2004; Ray et al., 2020) reveal a general coherence pattern between surface growth rate anomalies of N_2_O, CFC‐11, and CFC‐12 and the QBO. However, periods during which the coherence was interrupted were noted in Ray et al. (2020), and remain unexplained, raising questions about QBO modulation versus other impacts on near‐surface tracer concentrations.

In this study, we quantify modeled lower stratospheric tracer advection and assess mechanisms for and disruptions to the modulation of intrusions of N_2_O and CFC‐11‐poor air from the stratosphere into the troposphere. We examine the lower stratospheric tracer advection using a transformed Eulerian mean (TEM) tracer budget and compare calculations from a WACCM 10‐member ensemble simulation, JRA‐55 reanalysis, ACE satellite measurements, and AGAGE measurements. Interannual timescales of 1‐ to 5 years are our focus, as in Ray et al. (2020). We identify the dynamical conditions under which modeled lower stratospheric advection is enhanced and explore seasonal phase locking/unlocking patterns between the QBO phase and advection. The unique contributions of this study are not only to evaluate coherence patterns between the QBO, lower stratospheric advection and near‐surface tracer concentrations, but also to make use of the 10‐member ensemble to explain this disruption in coherence in terms of variability from one member to another. Since the real world is the equivalent of a single model realization, the modeled range provides insight for the interpretation of observed behavior. Furthermore, the 28‐month repeating QBO cycle in the model ensemble members simplifies the understanding of the model variability by eliminating changes in the QBO cycle as a driver of model variability. The potential role of QBO variability in modulating tracer advection and near‐surface tracer concentrations is the goal of this study but determining specific physical mechanisms for the phase unlocking patterns lies beyond its scope.

This study is laid out as follows. In Section 2, we describe the model simulations, reanalysis, and observations used for our analysis and the removal of anthropogenic increases from tracer concentrations prior to all calculations. We also explain the calculations of the TEM tracer budget and the analysis of the advection term in the TEM budget. In Section 3, we lay out the general pattern of modulation of extratropical and high‐latitude tracer advection by the QBO, briefly discuss physical processes behind the QBO modulation of tracer advection and how the modulation is communicated downwards as air parcels are flushed from the stratosphere back into the troposphere. In Section 4, we explain disruptions to this general pattern in terms of phase unlocking between the QBO and annual cycle. In Section 5, we present our conclusions.

The overall aim of improving the interpretation of variations in the QBO modulation of near‐surface tracer concentrations here is to advance understanding of the stratospheric dynamical influence of N_2_O and CFC‐11, such that any observed increases in surface concentrations of these gases can be accurately and confidently attributed to dynamical and/or anthropogenic influences.

## 2 Methods

### 2.1 Observations, Reanalysis, and Model Output

We analyze free‐running (FR) simulations of the Whole Atmosphere Community Climate Model (CESM1 WACCM4) with a fully interactive atmosphere and ocean in a perturbed state near the period of maximum ozone losses, with fully interactive chemistry. This fully coupled chemistry climate model has a horizontal resolution of 1.9° latitude by 2.5° longitude and 66 vertical levels with a high top at 5.1 × 10^−6^ hPa (∼140 km) (Garcia, Smith, Kinnison, de la Cámara, & Murphy, 2017; Marsh et al., 2013). The simulation we analyze covers the period of relatively high ozone depletion (1995–2024) with greenhouse gases (GHGs) and ozone depleting substances (ODSs) following RCP6.0. This setup has a repeated cyclic 28‐month quasi‐biennial oscillation, no solar cycle or solar proton events, and updated sulfate area densities including 21st century volcanic eruptions (Mills et al., 2016; Neely & Schmidt, 2016). The model was spun up from a REF‐C2 Chemistry Climate Model Initialization simulation commencing in 1955. The run has 10 ensemble members with monthly mean output. Each ensemble member was initialized with slightly different initial air temperatures in 1995 (Kay et al., 2015; Stone et al., 2019), to capture the diversity of internal dynamical variability in the system. Mixing ratio surface boundary conditions were used for CFC‐11 and N_2_O in this simulation. Global annual average values were specified for the period of the ensemble simulation from 1995 to 2024 with no latitudinal gradient. These values are based on the SPARC Chemistry‐Climate Model Initiative (CCMI) (Eyring et al., 2013; Morgenstern et al., 2017). The CFC‐11 values were based on observations from 1995 to 2010; the post 2010 projections originated from WMO (2010). The N_2_O mixing ratio surface boundary conditions were based on observations from 1995 to 2005; post 2005 projections followed RCP6.0 (Meinshausen et al., 2011).

We also use zonal‐mean measurements of N_2_O and CFC‐11 concentrations from the Fourier Transform Spectrometer on board the Atmospheric Chemistry Experiment (ACE) satellite mission (Bernath, 2017). We use Version 4.1 of the data set, which is in 5° latitude bins from 87.5°N to 87.5°S and from an altitude of 5.5–28.5 km at a vertical resolution of 1 km. The data are binned by season (December–February, March–May, June–August, September–November). The data set used extends from March 2004 to August 2020. While ACE has variable sampling during certain seasons at certain latitudes, our calculations sum over latitudes polewards of the tropical width which mitigates any potential sampling bias (see Section 2.4). For velocities and temperatures, we use 6‐hourly outputs of the Japanese 55‐year Reanalysis product (JRA‐55) for the period over which ACE satellite data is available, 2004–2020 (Japan Meteorological Agency, 2013). For near‐surface tracer concentrations, we analyze the global mean tracer concentration monthly data set from the Advanced Global Atmospheric Gases Experiment (AGAGE) spanning the date range July 1978–March 2020 (Prinn et al., 2018; Prinn et al., 2022).

### 2.2 Removing the Long‐Term Increases From Tracer Concentrations in the Stratosphere and at the Surface

The long‐term increase in each tracer was removed prior to performing any calculations or analyses. As N_2_O abundances increase approximately linearly with time, its stratospheric abundances were linearly scaled following the method described in Shah et al. (2020). For both WACCM and ACE stratospheric concentrations the stratospheric scaled N_2_O abundances were calculated as follows,

$$N_{2}O_{\left\{strat,dt\right\}}^{*}=N_{2}O_{\left\{strat,dt\right\}}\times \left(1-\frac{dt}{N}\frac{f_{\left\{500mb\right\}}}{N_{2}O_{\left\{500mb\right\}}}\right)$$

where dt is the interval of time between concentration measurements, N is the total number of months of the record, $f_{\left\{500mb\right\}}$ is the linear trend of $N_{2}O_{\left\{500mb\right\}}$ concentrations over the period of record and $N_{2}O_{\left\{500mb\right\}}$ is the zonal‐mean, temporal mean of tracer concentrations averaged over 30°N–30°S at 500mb. N_2_O global mean surface concentrations 1985–2021 from AGAGE were removed using a piece‐wise linear fit with breakpoints in 1988, 1991, 1994, 2002, and 2012 (Figure S1 in Supporting Information S1). These breakpoints were chosen based on the goodness‐of‐fit values, which helped ensure that the de‐trended timeseries were evenly distributed around zero. AGAGE N_2_O concentrations data require breakpoints to detrend the data because they cover a long‐time period that is sensitive to changes in surface emissions of trace gases.

As CFC‐11 abundances vary nonlinearly over the last three decades, their anthropogenic increases were removed differently to N_2_O. For CFC‐11 stratospheric concentrations in WACCM, the anthropogenic trend was removed by subtracting a second‐degree polynomial from CFC‐11 concentrations at each latitude (for model output, this subtraction was instead carried out at each longitude) and each stratospheric pressure level. For ACE stratospheric concentrations from March 2004 to August 2020, a piecewise linear trend was removed, with a breakpoint in 2012 following results from Montzka et al. (2018). For AGAGE near surface concentrations, a piecewise linear trend with breakpoints in 1989, 1991, 1993, 1995, 2002, and 2011 was used as a tangent approximation to CFC‐11's anthropogenic increase and its Montreal Protocol‐induced decrease of concentrations (Figure S1 in Supporting Information S1). Similar to the N_2_O AGAGE de‐trending, the breakpoints were identified based on the goodness‐of‐fit values.

### 2.3 Transformed Eulerian Mean Tracer Budget

The temporal, local change of abundances (ppb/day) of an atmospheric tracer $\bar{\chi }$ averaged over all longitudes (i.e., the zonal‐mean, denoted by an overline) can be separated into contributions from transport and chemistry using a Transformed Eulerian Mean (henceforth referred to as TEM) framework (Andrews et al., 1987) as follows

(1) $${\bar{\chi }}_{t}=-{\bar{v}}^{*}{\bar{\chi }}_{y}-{\bar{w}}^{*}{\bar{\chi }}_{z}+e^{z/H}\nabla \cdot M+\bar{S}$$

where subscripts represent partial derivatives, an overline represents the Eulerian zonal‐mean and a prime represents the deviation from the Eulerian zonal‐mean. The first two terms represent the meridional and vertical components of advection due to the residual circulation, where ${\bar{v}}^{*}$ and ${\bar{w}}^{*}$ are the meridional and vertical components of the residual mean meridional circulation, defined as (Andrews & McIntyre, 1976),

$${\bar{v}}^{*}=\bar{v}-e^{\frac{z}{H}}{\left(e^{-z/H}\frac{\bar{v^{\prime }\theta^{\prime }}}{{\bar{\theta }}_{z}}\right)}_{z}$$

(2) $${\bar{w}}^{*}=\bar{w}+\frac{1}{a\cos\phi }{\left(\cos\phi \frac{\bar{v^{\prime }\theta^{\prime }}}{{\bar{\theta }}_{z}}\right)}_{\phi }$$

in which a is the Earth's radius, ϕ represents latitude, H is the scale height typically used in the stratosphere (7 km, e.g., Abalos, et al., 2020), and $\bar{\theta }$, $\bar{v}$, and $\bar{w}$ are the Eulerian zonal‐mean potential temperature and meridional and vertical velocities respectively. The third term in Equation 1 represents the horizontal and vertical components of irreversible quasi‐horizontal isentropic eddy mixing, where the meridional and vertical components of the eddy flux vector M are defined as

$$M^{\left(y\right)}=-e^{-z/H}\left(\bar{v^{\prime }\chi^{\prime }}-\frac{\bar{v^{\prime }\theta^{\prime }}{\bar{\chi }}_{z}}{{\bar{\theta }}_{z}}\right)$$

(3) $$M^{\left(z\right)}=-e^{-z/H}\left(\bar{w^{\prime }\chi^{\prime }}+\frac{\bar{v^{\prime }\theta^{\prime }}{\bar{\chi }}_{y}}{{\bar{\theta }}_{z}}\right)$$

The last term in Equation 1, $\bar{S}$, is the net rate of change due to chemistry and is defined as the difference between the zonal‐mean chemical production rate, $\bar{P}$, and zonal‐mean chemical loss rate, $\bar{L}$, such that $\bar{S}=\bar{P}-\bar{L}$.

The role of each term can be examined by writing out the gradient of the eddy flux vector M and grouping terms together by component (Minganti et al., 2020),

(4) $${\bar{\chi }}_{t}=A_{y}+M_{y}+A_{z}+M_{z}+\left(\bar{P}-\bar{L}\right)-\bar{\epsilon }$$

where each term is

$$A_{y}=-{\bar{v}}^{*}{\bar{\chi }}_{y}$$

$$M_{y}=e^{z/H}\frac{1}{\cos\phi }{\left(M^{\left(y\right)}\cos\phi \right)}_{y}$$

$$A_{z}=-{\bar{w}}^{*}{\bar{\chi }}_{z}$$

(5) $$M_{z}=e^{z/H}{\left(M^{\left(z\right)}\right)}_{z}$$

such that $A_{y}$ represents the meridional component of residual advection, $M_{y}$ represents the horizontal transport of eddy mixing, $A_{z}$ represents the vertical component of residual advection, and $M_{z}$ represents the vertical transport of eddy mixing (Minganti et al., 2020). Each term has units of ppb/day. Therefore, $A_{y}$ and $A_{z}$ represent the contributions of meridional and vertical residual advection to the temporal rate of change of tracer concentrations, ${\bar{\chi }}_{t}$. Notably, Equation 4 does not represent a clean separation of advection and mixing and there exist some contributions to the advective transport which are not resolved by the residual advection. Consequently, the total mixing, $M_{y}+M_{z}$ includes not only the contribution from irreversible eddy mixing, but also the contribution from this unresolved advective transport (Andrews et al., 1987; Holton, 2004).

The final term ϵ in Equation 4 is the residual term which is the difference between ${\bar{\chi }}_{t}$ and the sum of the transport and chemical terms. Reasons for a non‐zero residual term in WACCM include the diagnostic variable used for TEM calculations that is not used to advect tracers because the model uses a finite volume dynamical core (Lin, 2004), implicit numerical diffusion (Conley, 2012), and interpolation of variables from hybrid sigma coordinates to pressure coordinates (Minganti et al., 2020).

Our calculations of the advection and mixing terms in the TEM tracer budget in Equation 5 were performed on variables in pressure coordinates. We calculated derivatives using a central finite difference scheme. We used backward finite difference on the upper horizontal boundary and along the northernmost poleward latitude and forward finite differences on the lower horizontal boundary and along the southernmost poleward latitude. Calculations on JRA‐55 reanalysis were performed on 6‐hourly snapshots and calculations on free‐running WACCM output were done on monthly‐mean data. As our calculations from WACCM were performed on coarser temporal resolution data, we validated them by reproducing results of the TEM tracer budget from Minganti et al. (2020) and found consistent results (not shown).

### 2.4 **Analysis of the Advective TEM Term** ${A_{y}+A}_{z}$ **in the TEM Tracer Budget**

This study primarily focuses on the advection of tracers in the extratropics and at high latitudes. The meridional and vertical advection through isentropes, ${A_{y}+A}_{z}$, is stronger than the horizontal and vertical transport by eddy mixing, $M_{y}+M_{z}$, because quasi‐horizontal isentropic eddy mixing is strong along isentropes and weak across isentropes. This motivates our focus on the meridional and vertical advection terms, ${A_{y}+A}_{z}$, in this study.

For all analyses presented in this study, we focus on the interannual variability that is defined by subtracting the 5‐year running mean of a given timeseries (e.g., meridional and vertical advection or tracer concentrations) from its 1‐year running mean. Specifically, the interannual anomaly of $A_{z}=-{\bar{w}}^{*}{\bar{\chi }}_{z}$ is calculated as follows

(6) $${\left(A_{z}\right)}_{interannualanomaly}={\left(A_{z}\right)}_{1yearrunningmean}-{\left(A_{z}\right)}_{5yearrunningmean}$$

and the interannual anomaly of $A_{y}=-{\bar{v}}^{*}{\bar{\chi }}_{y}$ is calculated as follows

(7) $${\left(A_{y}\right)}_{interannualanomaly}={\left(A_{y}\right)}_{1yearrunningmean}-{\left(A_{y}\right)}_{5yearrunningmean}$$

This follows the approach taken in Ray et al. (2020) and allows us to study the variability on 1‐ to 5‐year timescales. The results in this study are robust to changes in the details of the filter. Henceforth, the interannual anomaly subscript is dropped and Equations 6 and 7 are referred to as the interannual anomalies of $A_{y}$ and $A_{z}$.

We define the width of the tropical region as reflected in tracer concentrations using the gradient‐weighted latitude (GWL) approach of Shah et al. (2020). Tropical widths from N_2_O are calculated as functions of latitude, pressure and time using the GWL method for the width of the stratospheric tropics from tracer concentrations (Shah et al., 2020),

(8) $$\phi_{i}=\frac{\sum_{j=Eq}^{j=Pole}\phi_{j}{\left(\partial_{\phi }\chi \right)}_{ij}\cos\phi_{j}}{\sum_{j=Eq}^{j=Pole}{\left(\partial_{\phi }\chi \right)}_{ij}\cos\phi_{j}}$$

where i indexes over longitudes and j indexes over latitudes. The GWL metric has been shown to robustly calculate tropical widths from zonal‐mean data, allowing us to confidently apply it to zonally averaged ACE satellite measurements.

**Figure 1:**
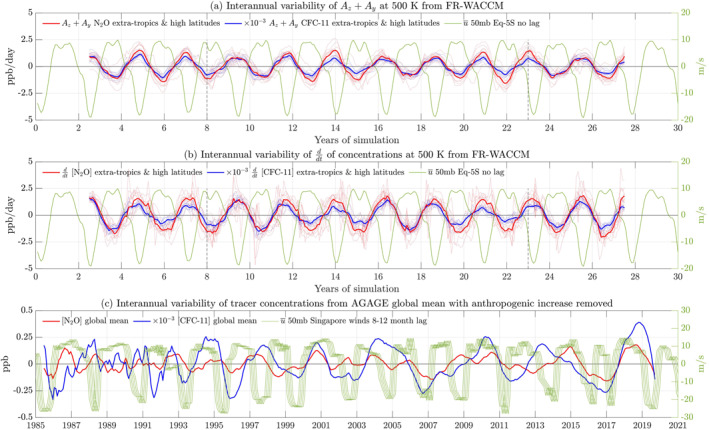
(a) The interannual variability of the meridional component plus the vertical component of advection ($A_{y}+A_{z}$) in the tracer TEM budget (defined as its 5‐year running mean subtracted from its 1‐year running mean) for N_2_O (red) and CFC‐11 (blue) in the Southern Hemisphere. $A_{y}+A_{z}$ is interpolated to the 500 K isentrope and horizontally summed along this isentrope from the tropical width to the pole. Individual ensembles are plotted in thin lines and the ensemble mean with a thick line. The equatorial zonal‐mean zonal wind $\bar{u}$ at 50 mb is plotted in green. (b) Same as Figure 1a but for the time derivative of tracer concentrations in the Southern Hemisphere with anthropogenic increase removed. (c) Same as Figure 1a but for the global‐mean of near‐surface N_2_O (red) and CFC‐11 (blue) concentrations from AGAGE with anthropogenic increase removed as described in Section 2.2. The Singapore zonal‐mean zonal winds at 50 mb (green) are underlaid with an 8–12 month lag.

In Figures 1, 3, 4, 5, the tropical widths and the advection terms of the TEM tracer budget are interpolated to the 500 K isentrope. The interannual anomalies of the meridional component of advection, $A_{y}$, and the vertical component of advection, $A_{z}$, along the 500 K isentrope (and in Section 4.4, the 350 K isentrope) are horizontally summed polewards from the tropical width to the pole, quantifying the total interannual isentropic tracer advection.

## 3 On the Imprint of the QBO on Near‐Surface Tracer Concentrations

In this section, we discuss how the meridional and vertical advection of trace gases is modulated by the QBO in the model and how this influence extends downwards to affect surface tracer concentrations.

### 3.1 Patterns of QBO Modulation of Stratospheric Advection $A_{y}+A_{z}$ on Interannual Timescales

We horizontally sum the interannual variability of $A_{y}+A_{z}$ polewards from the tropical width (identified using the GWL method) to the poles, thereby calculating the total extratropical, mid‐ and high‐latitude meridional and vertical tracer advection in ppb/day, as discussed in Section 2.4. The resulting $A_{y}+A_{z}$ timeseries at 500 K in the Southern Hemisphere is presented in Figure 1a. We choose to present these calculations at 500 K because it is the isentrope closest to 50 mb, where the QBO is typically defined, and, being in the lower stratosphere, is an indication of the interannual tracer advection that will eventually be transported across the tropopause and into the troposphere. The maxima of the $A_{y}+A_{z}$ timeseries occur during the easterly QBO phase and the minima occur during the westerly QBO phase (Figure 1a).

**Figure 2:**
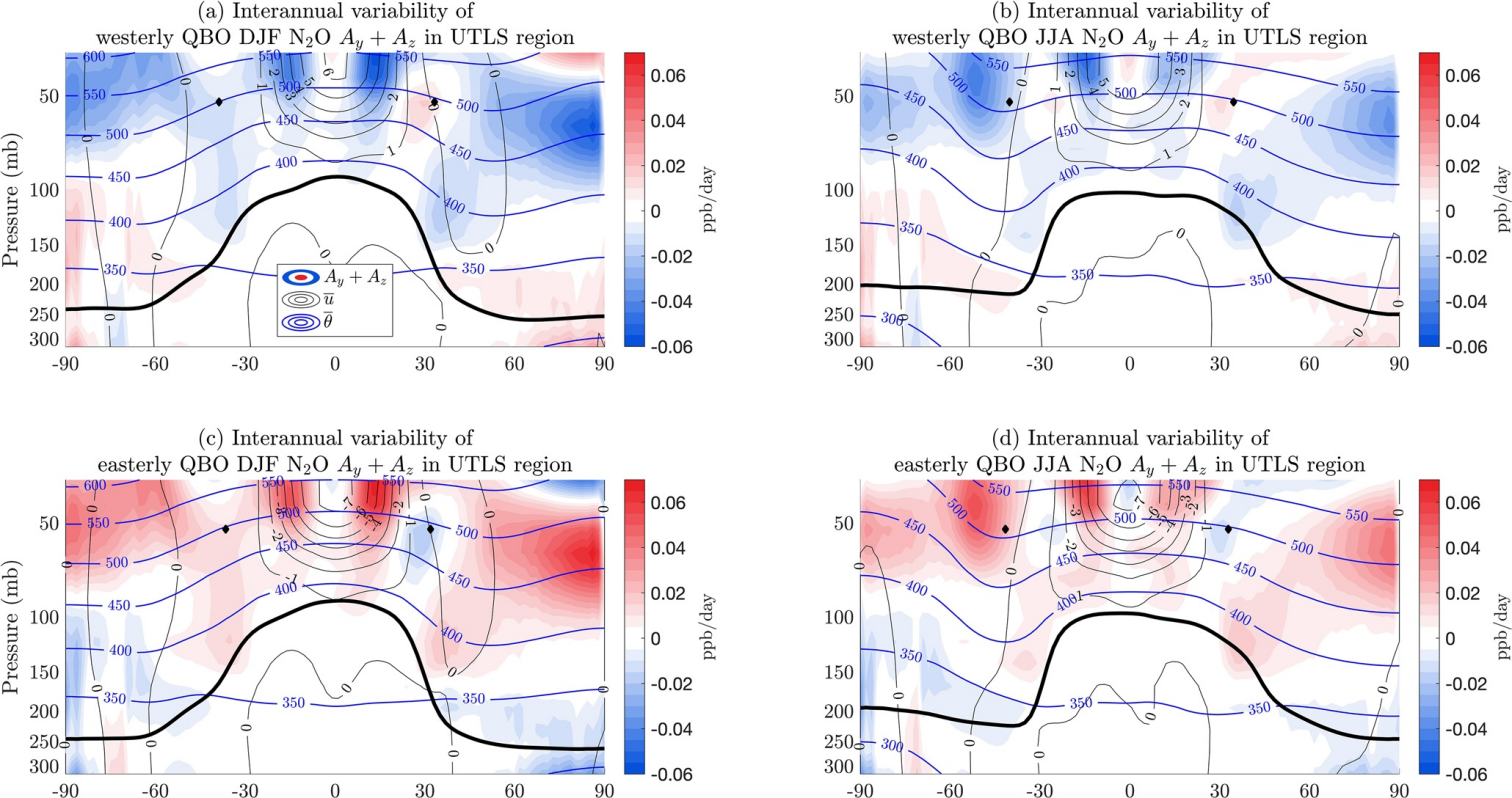
The sum of the interannual anomalies of the advection terms in the N_2_O TEM budget, $A_{y}+A_{z}$, plotted as a function of latitude and pressure on a log10 scale in the lower stratosphere. Each panel shows a seasonal average (DJF or JJA) for a given QBO phase: (a) westerly QBO in DJF, (b) westerly QBO in JJA, (c) easterly QBO in DJF, (d) easterly QBO in JJA. The thick black line in each panel marks the tropopause as defined in WACCM output. The tropical edges at the pressure level closest to the 500 K isentrope are plotted in black diamonds in each panel. A similar plot for CFC‐11 is in Figure S2 in Supporting Information S1.

To explain the occurrence of positive interannual tracer advection anomalies during the easterly phase, we consider dominant influences of the large‐scale stratospheric tracer advection on interannual timescales. We composite the ensemble‐mean interannual variability of $A_{y}+A_{z}$ by season (summer or winter) and by QBO phase in Figure 2. We plot the DJF (Figures 2a, 2c for N_2_O and Figures S2a, S2c in Supporting Information S1 for CFC‐11) and JJA (Figures 2b, 2d for N_2_O and Figures S2b, S2d in Supporting Information S1 for CFC‐11) averages of the interannual variability of the meridional and vertical advection terms, $A_{y}+A_{z}$, for the westerly QBO (Figures 2a, 2b, Figure S2a, S2b in Supporting Information S1) and easterly QBO (Figures 2c, 2d, Figures S2c, S2d in Supporting Information S1), and overlay it with arrows representing the meridional and vertical TEM velocities, $\left({\bar{v}}^{*},{\bar{w}}^{*}\right)$. We see strong positive interannual tracer advection anomalies in the easterly phase in both seasons, both hemispheres and for both tracers, with negative interannual anomalies in the westerly phase, again in both seasons, both hemispheres and for both tracers. This reinforces the result from Figure 1a that interannual anomalies of ${A_{y}+A}_{z}$ are positive during the easterly and negative during the westerly phase on 1‐ to 5‐year timescales.

The occurrence of positive interannual meridional and vertical advection anomalies during the easterly phase and negative interannual advection anomalies during the westerly phase can be partly explained by considering the sign of residual velocities and tracer gradients. We note that the vertical advective term is stronger than the meridional advective term at mid‐ to high‐latitudes, and therefore focus this discussion on the sign of the vertical advection term, $A_{z}$. In general, throughout the extratropical and mid‐to high‐latitude stratosphere, the vertical gradient of N_2_O and CFC‐11 is negative, that is, $\partial_{z}\chi <0$. At 50 mb, ${\bar{w}}^{*}$ associated with easterly minus westerly QBO composites is positive (Pahlavan, Fu, Wallace, & Kiladis, 2021). Therefore, during the easterly phase of the QBO, $A_{z}\left(=-{\bar{w}}^{*}\partial_{z}\chi \right)$ is positive and during the westerly phase of the QBO $A_{z}$ is negative (Figure 1a).

Our result of positive (negative) interannual advection anomalies during the easterly (westerly) phase in Figure 1a is consistent with the N_2_O QBO composites in figure 1 of Strahan et al. (2015). In these composites, the positive subtropical N_2_O anomaly (with respect to the 10‐year mean) during the easterly phase is influenced by tropical upwelling and advection of air out of the tropics into the subtropics by the QBO‐induced secondary MMC. The negative subtropical N_2_O anomaly during the westerly phase is influenced by extratropical downwelling of N_2_O‐poor air.

### 3.2 QBO Modulation of Lower Stratospheric Tracer Advection

While a comprehensive exploration of physical mechanisms behind the QBO's remote influence on the advective terms of a tracer's TEM budget is beyond the scope of this paper, in this subsection we briefly discuss the known key physical processes, specifically, the secondary mean meridional circulation (MMC) induced by the QBO and the teleconnection between the easterly QBO phase and the boreal winter polar vortex (the Holton–Tan effect; Holton & Tan, 1980, 1982).

The secondary MMC induced by the QBO phase consists of equatorial vertical motion and associated meridional divergence that drives convergence and divergence of tracer abundances (Choi et al., 2002; Plumb & Bell, 1982; Reed, 1962; Ribera et al., 2004; Wallace, 1967). Stronger downwelling occurs in the tropics in the westerly QBO shear zone than in the easterly shear zone (Baldwin et al., 2001, and references therein). Specifically, equatorial easterly shear zones are in thermal wind balance with a cold equatorial temperature anomaly, maintained by ascent. Downwelling in the extratropics induces a warm off‐equatorial temperature anomaly and balances the ascent. Conversely, in the westerly QBO shear zone the warm equatorial temperature anomaly is maintained by tropical descent which is balanced by off‐equatorial ascent (inducing a cold anomaly), as shown in Supplementary Movie 1 (consistent with previous literature e.g., Baldwin, et al., 2001; Choi et al., 2002; Ribera et al., 2004).

On the one hand, the extratropical descent induced by the lower stratospheric MMC during the easterly QBO shear zone strengthens the descending branch of the large‐scale Brewer Dobson circulation, and drives a stronger large‐scale circulation, while the extratropical ascent induced during the westerly shear zone weakens the descending branch of the BDC. That said, in the deep tropics, the largest anomalies in residual vertical velocity ${\bar{w}}^{*}$ and temperature (and tracer concentrations, as discussed in Section 3.1) occur during the easterly and westerly shear zones of the QBO and not during the easterly and westerly QBO phases (Pahlavan, Fu, Wallace, & Kiladis, 2021; Ribera et al., 2004). However, at extratropical latitudes, meridional propagation leads to the largest ${\bar{w}}^{*}$ and temperature interannual anomalies during the QBO phases and not during QBO phase transitions (Supplementary Movie 1).

On the other hand, additional physical processes interplay with the lower cell of the secondary MMC to influence strong advection in the easterly relative to the westerly phase. The MMC has an upper cell induced by the phase of the equatorial zonal‐mean zonal winds at 10 hPa, where $\bar{u}$ has the opposite sign compared to $\bar{u}$ at 50 hPa. The upper cell circulates in the opposite direction to the MMC cell in the lower stratosphere, using similar reasoning about thermal wind and meridional convergence/divergence as discussed earlier in this subsection (e.g. Choi et al., 2002; Ribera et al., 2004). Therefore, it is probable that the upper and lower cells of the MMC modulate the shallow and deep branches of the BDC simultaneously during any given QBO phase (e.g. Pahlavan, Fu, Wallace, & Kiladis, 2021). However, the extent to which the QBO phase in the mid‐upper stratosphere influences extratropical to high‐latitude tracer advection in the lower stratosphere is an area of ongoing research.

Several studies have noted that the QBO‐induced MMC extends into the extratropics and mid‐latitudes during the winter (Baldwin et al., 2001; Gray et al., 2001; Kinnersley & Tung, 1998) rather than being confined to 30°S–30°N tropical latitudes originally proposed by Plumb and Bell (1982). This seasonal asymmetry in tracer transport is due to the seasonal hemispheric asymmetries of the BDC's poleward branch and Rossby wave driving, both of which are stronger in the winter (Holton, 1989). The latitudinal extension of the secondary MMC can only occur in the presence of Rossby wave breaking, which occurs in the stratospheric winter due to the prevalence of westerly winds at extratropical‐ and mid‐latitudes. Prevailing westerlies permit vertical Rossby wave propagation into the stratosphere and subsequent wave breaking (Hitchman & Huesmann, 2007). Consequently, poleward tracer transport by the QBO‐induced MMC is stronger in the winter hemisphere than the summer hemisphere (Randel & Wu, 1996).

The QBO also indirectly modulates the strength of the BDC via its interactions with the wintertime polar vortex in the Northern Hemisphere. During the easterly QBO phase in boreal winter, this teleconnection between the polar vortex and the QBO, the Holton–Tan mechanism, induces a weaker boreal polar vortex and a stronger BDC (Holton & Tan, 1980, 1982). Garfinkel et al. (2012) probe two mechanisms for the weakening boreal polar vortex during the easterly QBO. The first is related to the $\bar{u}=0$ critical line which moves polewards due to enhanced convergence of Eliassen‐Palm flux in the subtropical lower stratosphere, extending the region of tropical easterlies. This is consistent with the Holton–Tan mechanism. The second is related to the lower stratospheric MMC extending polewards in winter, reducing the equatorward propagation of planetary waves from subpolar latitudes to midlatitudes, leading to enhanced planetary wave convergence in the polar region which weakens the upper polar vortex. They find that the mechanism associated with the MMC is more crucial to weakening the vortex than the mechanism associated with the critical line. In the context of this study, the importance of the QBO‐induced MMC's modulation of the boreal winter polar vortex (in addition to the Holton–Tan mechanism) adds nuance to the explanation of positive interannual tracer advection seen in the easterly QBO compared to the westerly QBO (Figure 1a) and of seasonal patterns in interannual advection in the Northern Hemisphere (Figures 2c, 2d, Figure S3c, S3d in Supporting Information S1 and Movie S1 in Supporting Information S2). The Holton–Tan mechanism does not apply to the Southern Hemisphere; the modulation of the Southern Hemisphere vortex by the QBO is sensitive to the QBO phase at ∼ 25 hPa (Baldwin & Dunkerton, 1998).

### 3.3 QBO Influence on Stratospheric and Near‐Surface Tracer Concentrations

We now consider the impact of positive stratospheric extratropical to high‐latitude interannual advection during the easterly QBO on the temporal rate of change of stratospheric tracer concentrations and on near‐surface concentrations on 1‐ to 5‐year timescales. We plot the interannual variability of the time derivative of N_2_O and CFC‐11 stratospheric concentrations horizontally summed from the extra‐tropics to the pole at 500 K as calculated from the 10 members in the WACCM simulations and overlay them with the zonal‐mean zonal winds at 50 mb (Southern Hemisphere calculations in Figure 1b). Vertical dashed black lines are plotted in Figures 1a and 1b to facilitate comparisons between the interannual advection anomalies and temporal change of tracer anomalies. We explain the QBO influence on tracer concentrations by following a parcel of air as it is transported from the upper stratosphere (poleward of the extratropics) down into the lower stratosphere, across the tropopause and into the troposphere.

Poleward of the extratropics, air in the upper stratosphere is generally tracer‐poor compared to air in the lower stratosphere. Interannual tracer advection during the easterly shear zone therefore brings down tracer‐poor air, diluting tracer abundances in the lower stratosphere, compared to interannual tracer advection during the westerly shear zone which brings down less tracer‐poor air compared to the easterly shear zone. There is also a horizontal transport lag timescale as the air is transported laterally in the extratropics and then downwards at mid‐to‐high latitudes (see Section 5.1). By the time the strongest part of the easterly QBO phase (i.e., most negative $\bar{u}$) begins, the horizontal and vertical lags have transported the tracer‐poor air into the lower stratosphere. Consequently, the interannual $\partial_{t}\chi$ stops increasing as tracer‐poor air accumulates and begins decreasing as tracer abundances are diluted by strong downward advection of tracer‐poor air during the easterly phase (Figures 1a, 1b). Conversely, in the transition from easterly to westerly winds, less tracer‐poor air is brought down from the upper stratosphere as the advection weakens and, consequently, the interannual variability of concentrations begins increasing during the strongest part of the westerly phase (that is, most positive $\bar{u}$).

Furthermore, based on Equation 4, we expect the interannual variability of the advective terms to closely track the variability in the temporal change of tracer concentrations, consistent with $A_{y}+A_{z}$ representing the residual advective contributions to $\partial_{t}\chi$. Interannual $\partial_{t}\chi$ at 500 K maximizes in the easterly phase and minimizes in the westerly phase (Figure 1b), indicating that the tracer concentrations themselves peak during phase transitions (i.e., the shear zones) of the QBO. Specifically, as the westerly phase ends and the easterly phase begins, interannual $\partial_{t}\chi$ increases and, as the easterly phase ends and the westerly phase begins, interannual $\partial_{t}\chi$ decreases.

As stratospheric air downwells at extratropical and at high latitudes, it is transported across the tropopause and is flushed into the troposphere (Figure 2 and Figure S2 in Supporting Information S1). To relate the impact of this flushing to tropospheric tracer concentrations on 1‐ to 5‐year timescales, we plot the interannual variability of the globally averaged near‐surface concentrations from AGAGE stations in Figure 1c. The equatorial Singapore zonal‐mean zonal winds at 50 mb, representing QBO variability, is lagged by 8–12 months to enable a comparison between $\bar{u}$ and near‐surface concentrations, which is the range of timescales on which air is transported from the lower stratosphere to the surface (Ray et al., 2020; Ruiz et al., 2021). The near‐surface concentrations generally have their lowest values during the easterly phase and their largest values during the westerly phase. As N_2_O and CFC‐11 are predominantly depleted in the stratosphere, the stratospheric air being flushed into the troposphere has lower abundances of these gases compared to tropospheric air and therefore tropospheric concentrations are diluted.

The dilution is stronger during the easterly phase and more tracer‐poor air is flushed into the troposphere than during the westerly phase. Therefore, the westerly phase of the QBO that corresponds to weak dilution of tracer concentrations is the phase during which near‐surface concentrations generally maximize (Figure 1c). In contrast, the easterly QBO phase corresponds to strong stratosphere‐troposphere advection and consequently to strong dilution of tropospheric abundances (Ruiz & Prather, 2022). There are other influences on near‐surface concentrations besides the lower stratospheric tracer anomaly, such as seasonal stratosphere‐troposphere mass exchange; however, these fall beyond the scope of this study.

However, there are important departures from this general QBO phase‐based pattern in both individual members of the ensemble and in the observations in the stratosphere and at the surface. For instance, in Figures 1a and 1b, a number of the individual WACCM ensemble members (plotted as thin lines) exhibit departures from the ensemble‐mean pattern of both $A_{y}+A_{z}$ and the time‐derivative of tracer concentrations maximizing in the easterly QBO and minimizing in the westerly QBO. Similarly, AGAGE near‐surface tracer concentrations, which generally maximize during the westerly QBO phase and minimize during the easterly QBO phase (Figure 1c), clearly depart from their general pattern on certain occasions. For example, the westerly phase in 2008 and 2012 have weaker interannual concentration anomalies than other westerly phases and the easterly QBO phase in 2009 and 2014 have stronger interannual concentration anomalies than other easterly phases. We further discuss such departures from the general pattern in the next section.

## 4 Phase Unlocking Patterns in the Modulation of the Stratospheric Interannual Advection Anomalies $A_{y}+A_{z}$ by the QBO

In this section, we focus on departures from the general patterns with respect to the QBO phase seen in lower stratospheric residual advection (${A_{y}+A}_{z}$ terms in Equation 4), in the rate of change of lower stratospheric tracer concentrations and in near‐surface tracer concentrations in calculations from both model output and observations.

The periodicity of the QBO is not exactly 24 months, hence the name Quasi‐Biennial Oscillation, nor are the durations of each QBO phase constant. Assuming that the length of a QBO cycle is ≈28 months, it will take ≈7 years for the QBO to return to its initial point in the annual cycle (Gray and Dunkerton, 1990). During this period, the phase relationship between the QBO and advective transport (and between the QBO and tracer concentrations; Gray & Dunkerton, 1990) continuously changes, particularly when the equatorial $\bar{u}$ is not regularly oscillating. In this section, we use the 10 ensembles of FR‐WACCM4 simulations to explore seasonal phase locking and unlocking patterns between the advection ($A_{y}+A_{z}$) and QBO phase.

**Figure 3:**
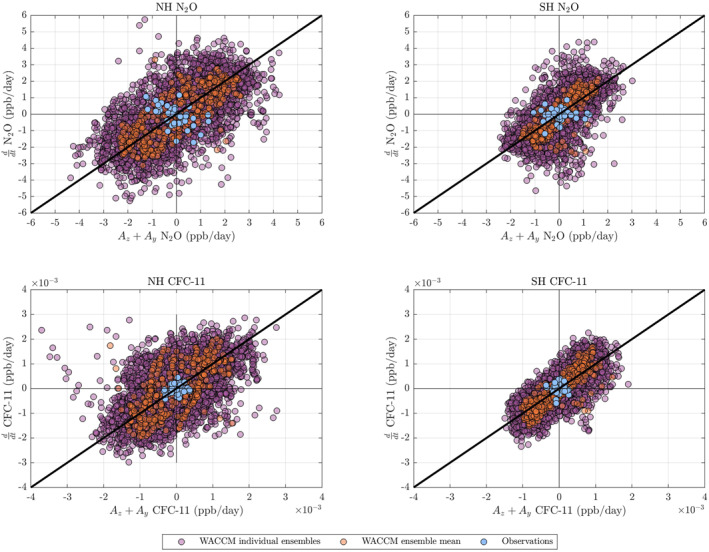
Scatter plots of ${A_{y}+A}_{z}$ (*x*‐axis, ppb/day) against the time derivative of tracer concentrations (*y*‐axis, ppb/day) at 500 K. The panels organize results by tracer and by hemisphere: (a) Northern Hemisphere ${A_{y}+A}_{z}$ of N_2_O against $\frac{d}{dt}$ (N_2_O), (b) Southern Hemisphere ${A_{y}+A}_{z}$ of N_2_O against $\frac{d}{dt}$ (N_2_O), (c) Northern Hemisphere ${A_{y}+A}_{z}$ of CFC‐11 against $\frac{d}{dt}$ (CFC‐11), (d) Southern Hemisphere ${A_{y}+A}_{z}$ of CFC‐11 against $\frac{d}{dt}$ (CFC‐11).

To explore whether the relationship between the advection and the time‐derivative of stratospheric tracer concentrations is consistent between individual model ensemble simulations and observations, we create a scatter plot for each tracer by hemisphere. Figure 3 plots the interannual anomalies of $A_{y}+A_{z}$ against the interannual anomalies of the time derivative of tracer concentrations, showing that for both N_2_O and CFC‐11, the inferences from observations fall within the envelope of the individual ensemble members. However, while N_2_O interannual advection and concentration anomalies are of the same order of magnitude in both observations and the model, CFC‐11 anomalies from observations are approximately one‐third less than calculations from the model, indicating that the model is more variable than the observations. This discrepancy may point to greater noise due to uncertainties in the observational data relative to the simulations, but complications due to removing a nonlinear anthropogenic increase from models and observations may also contribute for CFC‐11. Therefore, we largely focus on N_2_O only in the rest of the paper.

### 4.1 Phase Relationship Between QBO and 500 K Tracer Advection in Individual Ensemble Members

We explore the seasonal phase relationships of tracer advection by QBO phase in individual WACCM ensemble members. The QBO in this ensemble simulation is a regularly repeating 28‐month cycle, and therefore the results presented in this subsection are from a control scenario when the periodicity of the QBO does not vary over time. Each of the 10 ensemble members is a 30‐year simulation (1995–2024). We define the westerly QBO phase as above the 75th percentile of zonal‐mean zonal‐wind at 50 hPa from 5N to 5S and the easterly QBO phase as under its 25th percentile. This definition is because we are interested in how the advective contributions and concentrations behave when the QBO phase is clear rather than transitional, given that patterns exist between tracer advection ($A_{y}+A_{z}$) and QBO phase (Figure 1a). Therefore, we do not consider months when the equatorial zonal‐mean zonal winds are switching direction.

Additionally, we check that these instances of strong westerly and easterly QBO phases are not preferentially sampling El Niño or La Niña events by calculating the Niño3 index, defined as the area‐averaged sea surface temperature anomalies in the Niño3 region (5°S–5°N, 150°–90°W) following Rao and Ren (2016). We find this Niño3 index is close to zero across all ensembles during established westerly and easterly QBO phases (−0.07 in the Northern Hemisphere and −0.12 in the Southern Hemisphere).

**Figure 4:**
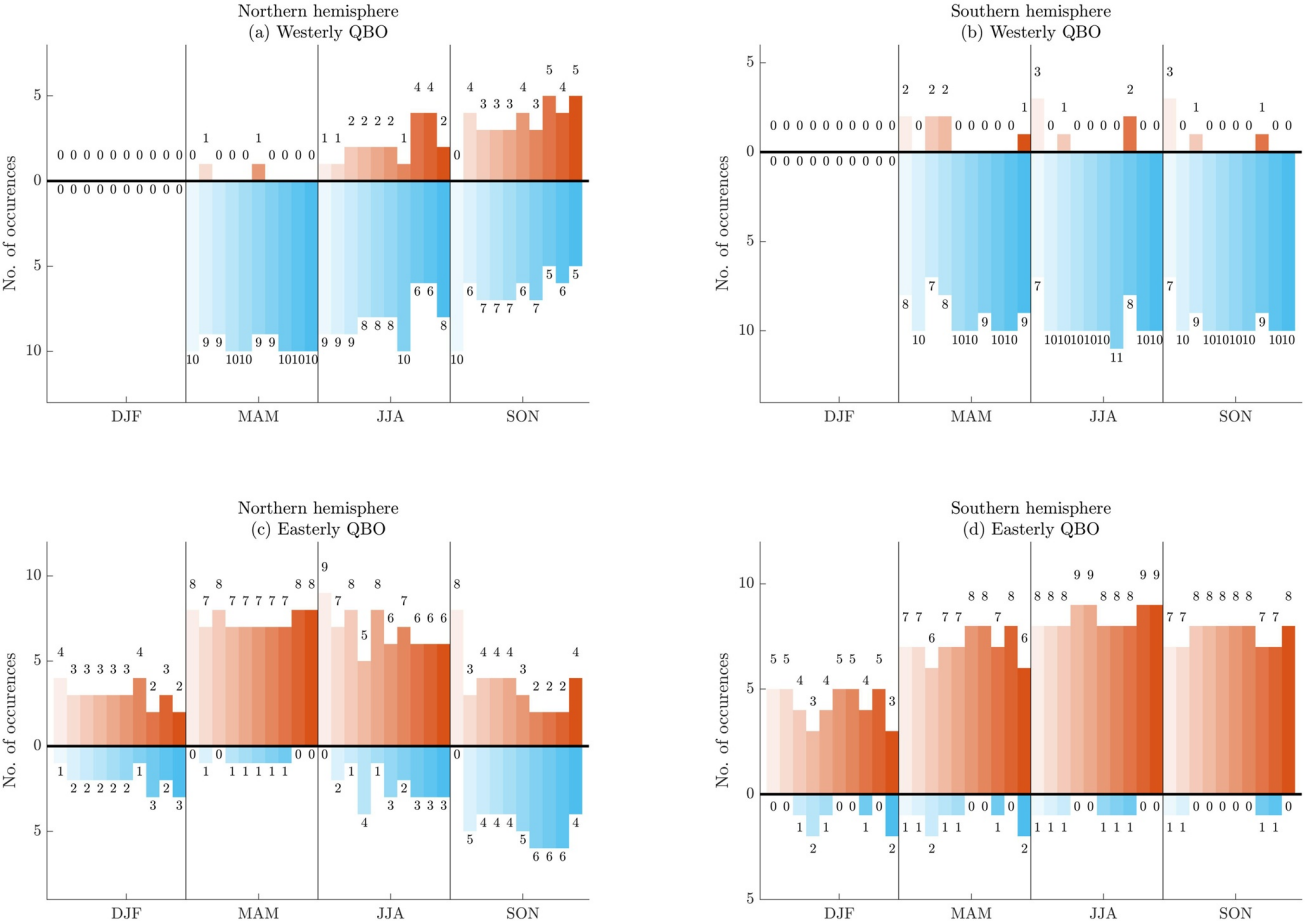
In each QBO phase, the number of seasonal occurrences of positive (orange) and negative (cyan) interannual anomalies (i.e., 1‐year minus 5‐year running means) of ${A_{y}+A}_{z}$ of N_2_O calculated at 500 K from WACCM, from each individual ensemble member are presented for each season, starting with ensemble member 1 in lighter shades and ending with ensemble member 10 in darker shades. The panels organize results by QBO phase and by hemisphere: (a) westerly Northern Hemisphere, (b) westerly Southern Hemisphere, (c) easterly Northern Hemisphere, (d) easterly Southern Hemisphere. Results for CFC‐11 are in Figure S3 in Supporting Information S1.

During these instances of established easterly and westerly QBO, we count the number of positive and negative occurrences of interannual tracer advection at 500 K calculated from each individual WACCM ensemble member by season and by hemisphere. We present each ensemble's counts in different shades of orange for positive anomalies and in different shades of blue for negative anomalies for each season, hemisphere and QBO phase (Figure 4). Figure S3 in Supporting Information S1 contains results for CFC‐11.

As expected from Figure 1a, we find that occurrences of negative anomalies in the westerly phase generally far outnumber occurrences of positive anomalies. In the easterly phase, occurrences of positive anomalies far outnumber occurrences of negative anomalies. This underscores the broad pattern of positive interannual tracer advection in the easterly phase compared to the westerly phase and is consistent with Figures 1, 2, 3 as well as with our discussion in Section 3.3.

### 4.2 Phase Locking and Unlocking Patterns Between QBO and 500 K $A_{y}+A_{z}$ From Model Simulations

We now discuss these results based on synchronizations (i.e. “locking”) and unsynchronizations (i.e., unlocking) of the phases of the annual cycle to the QBO. Said differently, when the relationship between the QBO phase and a seasonal timeseries of interest (e.g., tracer concentrations or tracer advection) is *systematic*, the annual cycle can be considered “phase locked” to the QBO. “Phase unlocking” occurs when the relationship between the QBO phase and this timeseries is variable and not seasonally systematic.

We note occurrences of “phase locking” when the relationship between the QBO phase and the advective terms $A_{y}+A_{z}$ is *systematic* based on season. For instance, the spring seasons in both hemispheres (i.e., MAM in the Northern Hemisphere and SON in the Southern Hemisphere) have an overwhelming majority of positive anomalies during the easterly QBO phase and an overwhelming majority of negative anomalies for the westerly QBO phase in all 10 ensemble members and for both N_2_O (Figure 4) and CFC‐11 (Figure S3 in Supporting Information S1). Spring in both hemispheres are examples of seasons when the QBO locks consistently to the annual cycle in the WACCM ensemble. These examples are consistent with previously published studies that find subtropical ozone anomalies in the winter‐spring of both hemispheres due to synchronizations between the seasonal cycle and the QBO (Gray & Dunkerton, 1990; Gray & Pyle, 1989). Phase locking during the Southern Hemisphere spring has been explained in the context of large zonal wavenumber 1 anomalies in the winter‐spring season which exhibit preferences for phase locking (Randel & Cobb, 1994).

We briefly place our lower stratospheric phase locking results from WACCM simulations in the context of previous studies that explored relationships between the QBO phase and annual cycle (Wallace et al., 1993; Hamilton & Hsieh, 2002). Months during which wind transitions are most likely can be identified from months when QBO descent is impeded. QBO descent rates slow down during months with high seasonal planetary wave activity that causes uplifting in the tropics (Coy et al., 2020). As a result of this stalling during strong wind shear, the phase of the QBO tends to spend longer in the easterly than the westerly wind phase (Coy et al., 2020; Fraedrich et al., 1993), providing a useful diagnostic tool for QBO structure and for phase relationships. That said, Coy et al. (2020) find little evidence for phase locking of the QBO to the annual cycle. We note a key distinction between our study and theirs: we explore seasonal phase relationships between the QBO and tracer advection, rather than examining the phase relationship between the amplitude and phase of the QBO, derived from empirical orthogonal functions of the zonal‐mean zonal wind, and the annual cycle of the equatorial zonal‐mean residual vertical velocity and meridional heat flux. Additionally, our WACCM simulations use a regularly repeating QBO cycle, while their MERRA‐2 Atmospheric Model Intercomparison Project ensemble using the Global Earth Observatory GCM 10‐member simulation do not.

“Phase unlocking,” on the other hand, occurs when the relationship between the QBO phase and the advective terms $A_{y}+A_{z}$ is variable and is not systematic based on season. In our calculations from the model, seasons with phase unlocking patterns exhibit deviations from the general pattern. (This general pattern exhibits positive (negative) interannual advection anomalies during the easterly (westerly) QBO phase, as discussed in Section 3.1.) Examples of seasons when the QBO influence on N_2_O advection unlocks from the annual cycle include summer, JJA, and fall, SON, in the Northern Hemisphere, when a substantial number of positive (negative) interannual advection anomalies during the westerly (easterly) QBO phase occur (Figure 4a for westerly QBO, Figure 4c for easterly QBO).

Strikingly, in DJF of both hemispheres, there are no instances of well‐established westerly QBO phases, related to the way the QBO happens to be initialized in this set of WACCM simulations with a regularly repeating 28‐month undisrupted QBO cycle. Hence, every DJF season, the zonal‐mean zonal wind is not in the 0.75 quantile nor in the 0.25 quantile and, consequently, occurrences of positive and negative interannual anomalies of advection of N_2_O and CFC‐11 are not captured by the choice of quantiles we adopt for diagnosing the phase of the QBO (Figures 4a, 4b, Figures S3a, S3b in Supporting Information S1).

By comparing seasonal phase relationships between the tracer advection of N_2_O and CFC‐11, we observe consistent relationships in the Southern Hemisphere and differing behavior in the Northern Hemisphere. In the Southern Hemisphere, the phase relationships between the QBO and the tracer advection of both N_2_O (Figures 4b, 4d) and CFC‐11 (Figures S3b, S3d in Supporting Information S1) are predominantly phase locked across all seasons; however, instances of phase unlocking do occur. On the other hand, in the Northern Hemisphere fall (SON) and summer (JJA) seasons, the frequency of phase unlocking of N_2_O advection exceeds the frequency of phase unlocking of CFC‐11 advection. Additionally, while CFC‐11 phase locking and unlocking patterns differ across hemispheres, phase unlocking in N_2_O advection occurs more often in the Northern than the Southern Hemisphere. Specifically, the phase unlocking in N_2_O advection occurring in boreal summer and fall is more frequent than the phase unlocking of N_2_O advection that occurs in austral summer and fall.

In the context of our calculations from the WACCM ensemble output, we suggest that these tracer‐tracer and hemispheric differences in phase unlocking frequency in the lower stratosphere are linked to where each tracer's stratospheric loss occurs and how the loss regions influence their advection by the lower and upper branches of the Brewer Dobson Circulation, respectively, in this model. Specifically, CFC‐11 loss occurs lower down in the stratosphere than N_2_O loss (∼25 km altitude compared to ∼32 km altitude, e.g., Ray, et al., 2020; Ruiz et al., 2021) and therefore is transported differently by the upper and lower BDC branches, for example, the lower branch of the BDC is more important for CFC‐11 stratospheric transport than it is for N_2_O. The lower stratospheric phase relationships between N_2_O and CFC‐11 in both hemispheres differ at least in part because of this effect. Furthermore, as the upper branch of the BDC is stronger in the Northern Hemisphere than in the Southern Hemisphere (Butchart, 2014), the phase relationships of N_2_O advection should be expected to differ by hemisphere.

The tracer‐tracer and hemispheric differences suggest that stratospheric tracer advection by the upper branch of the BDC is associated with a greater frequency of phase unlocking than advection by the lower branch of the BDC, and that stratospheric tracer advection by a strong circulation is associated with a higher frequency of phase unlocking than by a weak circulation. This may have implications for phase unlocking patterns in changing climate conditions under which the BDC is predicted to strengthen (e.g. Butchart et al., 2006; Butchart et al., 2010), the QBO amplitude is projected to decrease (Richter et al., 2020) and the QBO period is expected to shorten (DallaSanta et al., 2021; Garcia R. R., 2021).

### 4.3 Phase Relationships Between QBO and 500 K $A_{y}+A_{z}$ and Between QBO and Near‐Surface Tracer Concentrations From Model Simulations and Observations

**Figure 5:**
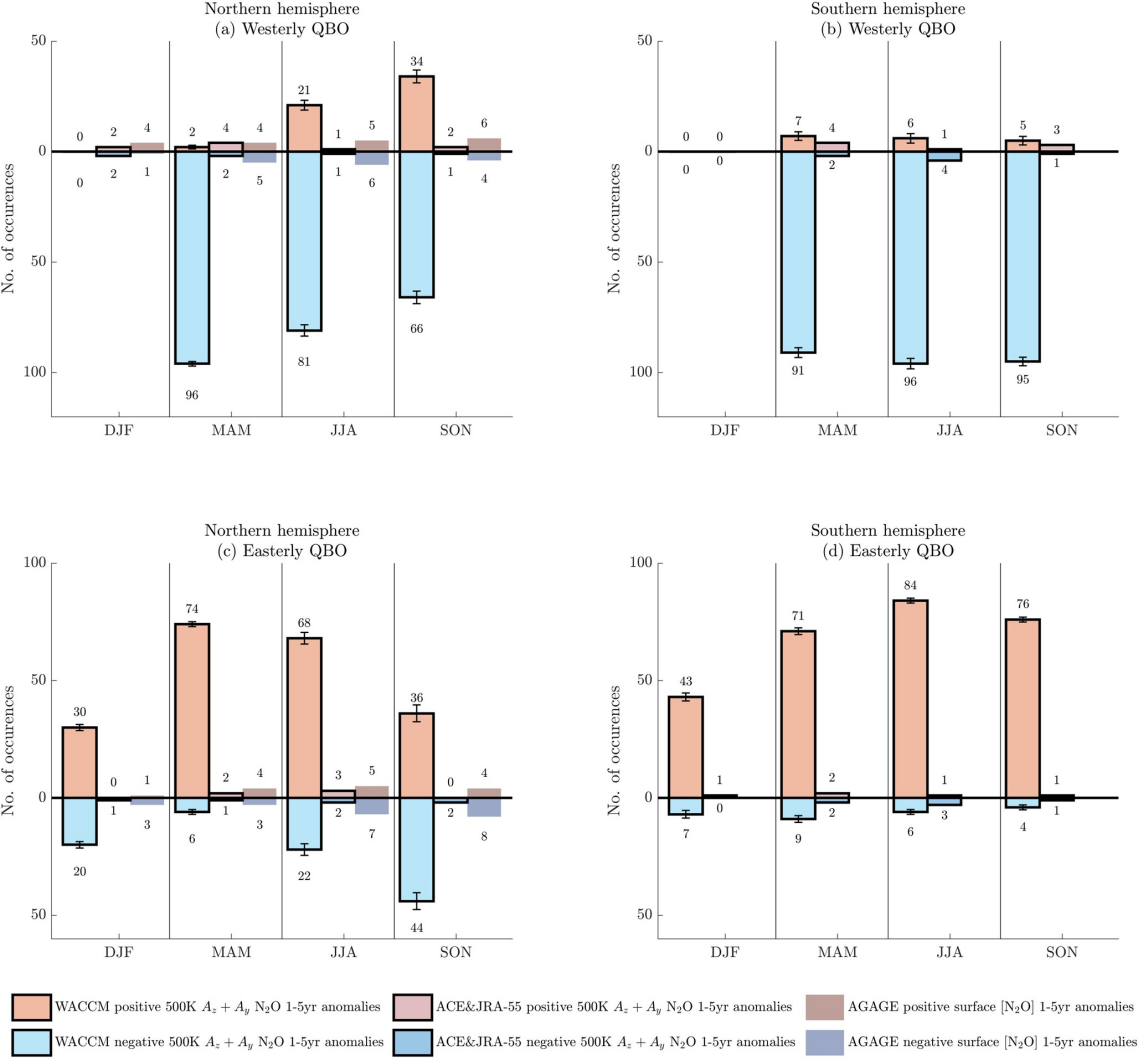
Same as Figure 4, but for cumulative results from WACCM compared to observations: (i) ${A_{y}+A}_{z}$ of N_2_O calculated at 500 K from WACCM presented as the cumulative counts of each ensemble member (orange/cyan), (ii) $A_{y}+A_{z}$ of N_2_O calculated at 500 K from JRA‐55 and ACE (red/blue) and (iii) global‐mean near‐surface tracer concentrations of N_2_O from AGAGE (maroon/purple without a black outline). The panels organize results by QBO phase and by hemisphere (with total counts in each quantile for model ensemble/ACE‐JRA55 results/AGAGE): (a) westerly Northern Hemisphere (300/15/35), (b) westerly Southern Hemisphere (300/15), (c) easterly Northern Hemisphere (300/11/35), (d) easterly Southern Hemisphere (300/11). The error bars on the WACCM results mark the 2 standard deviations of the individual ensemble results. Results for CFC‐11 are in Figure S4 in Supporting Information S1.

Next, we examine whether the seasonal phase relationships of the advective terms (${A_{y}+A}_{z}$) calculated from ACE tracer measurements and JRA‐55 reanalysis fall within the range of the seasonal phase locking patterns of ${A_{y}+A}_{z}$ calculated from the WACCM ensemble members. We count the instances of positive and negative interannual anomalies of ${A_{y}+A}_{z}$ from the sum over all 10 ensemble members by season and by QBO phase (defined in the same way as in Figure 4 and Figure S3 in Supporting Information S1) and from ACE and JRA‐55 stratospheric advection. The AGAGE surface concentration anomalies are partitioned into easterly and westerly phases based on the QBO phase 11 months earlier than the AGAGE measurements, using the same quantile QBO phase definitions as in Figure 4 and Figure S3 in Supporting Information S1. We also count the number of positive and negative interannual anomalies of the global mean of near‐surface AGAGE tracer concentrations by season and by hemisphere for N_2_O (Figure 5) and for CFC‐11 (Figure S4 in Supporting Information S1). The global‐mean near‐surface concentrations are plotted in the Northern Hemisphere panels of Figure 5 and Figure S4 in Supporting Information S1, as that is where the majority of AGAGE stations are. The 30‐year WACCM 10‐member ensemble has 1,200 (=30 × 4 × 10) seasons of simulation, while the stratospheric advection from observations has 66 seasons (2004–2020) and AGAGE station data has 96 seasons (1997–2020).

We find that in all but a few seasons, the behavior of the observations fall within the range of the individual ensemble members. Here, range is defined as the cumulative counts from each WACCM ensemble member and the two standard deviation calculated from individual ensemble results, marked by the error bars in Figure 5. For instance, during JJA and SON in the Northern Hemisphere, phase unlocking occurs in both the model and the observations for both N_2_O and CFC‐11 in both QBO phases. A notable exception to model‐observation agreement is DJF in the Northern Hemisphere of the westerly QBO (Figures 5a, 5b, Figures S4a, S4b in Supporting Information S1). As already noted, there are no instances of established QBO phases with zonal‐mean zonal winds in the upper or lower quantile in the model due to the timing of the initialization of the imposed regular 28‐month QBO cycle (Section 4.1). A comparison of the seasonal QBO phase partitioning between WACCM and JRA55 confirms that there are no westerly DJF occurrences in WACCM (Figure S5 in Supporting Information S1). In the observed atmosphere, on the other hand, the occurrences of positive and negative interannual anomalies of ${A_{y}+A}_{z}$ and tracer concentrations anomalies are linked to irregularities in the QBO cycle, resulting in strong easterly and westerly equatorial zonal‐mean zonal winds in all seasons. That said, it is remarkable that in the Southern Hemisphere during the westerly QBO there are no instances of strong nor weak N_2_O and CFC‐11 advection neither in the observations nor in the model.

The results from near‐surface N_2_O concentrations generally fall within the uncertainty range of the ensemble member results. We refrain from a detailed comparison between the ensemble results and measured concentrations as the model runs employ trace gas surface boundary conditions. As the QBO cycle is regular and repeating in WACCM, we do not expect the WACCM phase relationships in the lower stratosphere to completely capture those at the near‐surface from observations. Additionally, these near‐surface results indicate the complexity of linking lower stratospheric tracer advection to the concentrations at the near‐surface. Given that the observed record of near‐surface concentrations is longer than the observed record of stratospheric concentrations, the sample size of calculations in the lower stratosphere is smaller than at the near‐surface. This confounds efforts to use calculations from the observed lower stratosphere to explain the differences between WACCM and near‐surface results.

Studies that examine stratosphere‐troposphere exchange (STE) flux on the surface variability of N_2_O and CFC‐11 demonstrate that when the variation in STE flux is dominated by annual and QBO cycles, the phase and amplitude of surface variations are correspondingly influenced (Ruiz & Prather, 2022). Furthermore, variability in near‐surface N_2_O concentrations is associated with the STE flux and is driven by stratospheric loss on annual (Northern Hemisphere) and QBO (Southern Hemisphere) cycles (Ruiz D. J. et al., 2021; Ruiz & Prather, 2022). This study expands current literature examining stratospheric dynamical influences on surface trace gas variability by highlighting an additional association between lower stratospheric interannual tracer advection and interannual variability of near‐surface tracer concentrations via phase locking/unlocking from the QBO.

### 4.4 Phase Relationships Between QBO and 350 K Interannual Anomalies $A_{y}+A_{z}$ From Model Simulations and Observations

**Figure 6:**
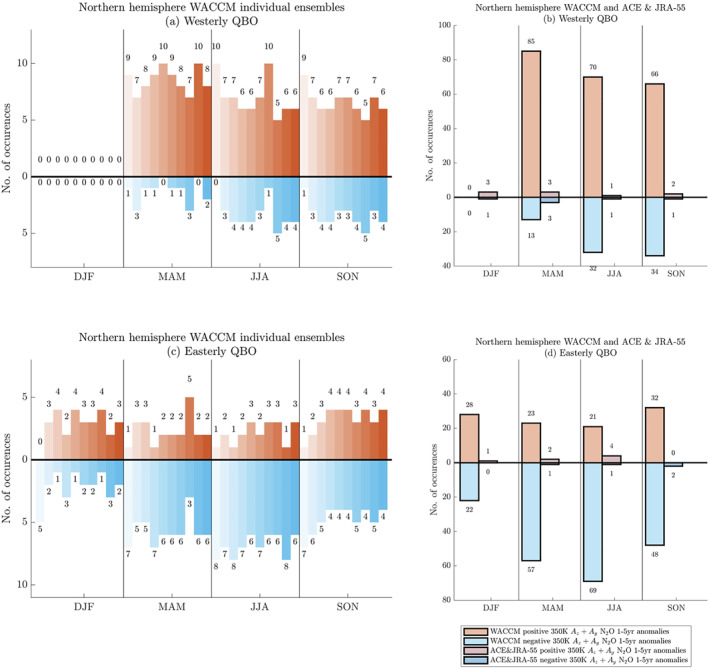
350 K results for interannual $A_{y}+A_{z}$ for N_2_O in the Northern Hemisphere with panels (a) and (c) similar to Figures 4a, 4c and panels (b) and (d) similar to Figures 5a, 5c.

To explore the seasonal phase relationships between 350K interannual advection anomalies and the QBO phase, we use the definitions of established easterly and westerly QBO phases to count the number of positive and negative occurrences of interannual N_2_O advection anomalies at 350K by season and by hemisphere. Northern Hemisphere results are plotted in Figure 6 and Southern Hemisphere results are plotted in Figure S6 in Supporting Information S1. In both figures, calculations are presented from each individual WACCM ensemble member (panels (a) and (c)) and also from ACE observations and JRA55 reanalysis (panels (b) and (d)). On comparing Figures 4 and 5 to Figure 6, the general phase locking pattern at 350 K is opposite to the pattern at 500 K and consistent with the analysis of Supplementary Movie 1. Physically, this opposite pattern is explained by a horizontal and vertical transport lag as the interannual anomalies are transported laterally and downwards. For instance, when the zonal‐mean zonal winds are westerly at the equator at 50 hPa, the 500 K surface receives the negative interannual advection anomalies from the current westerly phase and the 350 K surface receives the positive interannual advection anomalies from the *previous* easterly phase.

Specifically, during the westerly QBO phase, the systematic seasonal relationship between the QBO phase and the advective terms $A_{y}+A_{z}$ at 350 K generally indicates more positive than negative anomalies in MAM, JJA and SON for the ensemble members in both hemispheres (Figure 6a, Figure S6a in Supporting Information S1). However, certain ensemble members display phase unlocking patterns, for example, ensemble eight in both JJA and SON has five counts of positive and five counts of negative anomalies in both hemispheres of the westerly QBO. Ensemble 1 in Southern Hemisphere MAM has five counts of positive and five counts of negative anomalies in the westerly QBO.

During the easterly QBO phase, the systematic seasonal relationship between the QBO phase and the advective terms $A_{y}+A_{z}$ at 350 K indicates more negative than positive counts during the MAM and JJA seasons in the Northern Hemisphere and during MAM, JJA and SON seasons in the Southern Hemisphere (Figure 6c, Figure S6c in Supporting Information S1). Once again, there are individual ensemble members who display phase unlocking behaviors, such as ensemble 10 in DJF in both hemispheres with 3 positive counts and 2 negative counts. During DJF, the total count across all 10 ensembles is 28 positive counts to 22 negative counts in the Northern Hemisphere and 20 positive counts to 30 negative counts in the Southern Hemisphere (Figure 6b, Figure S6b in Supporting Information S1). Therefore, DJF is a season for which the WACCM ensemble results indicate phase unlocking is at least as frequent as phase locking in the Northern Hemisphere and is almost as frequent in the Southern Hemisphere.

Just as at 500 K, a comparison between the ensemble results and the results from ACE observations and JRA55 reanalysis is complicated because the observational results have far fewer counts than the WACCM ensemble and because the observed QBO is a nonregular and nonrepeating cycle. At 350 K, however, this comparison is further complicated because of the higher incidences of phase unlocking in the model ensembles.

The interannual anomaly of near‐surface tracer concentrations can be appropriately lagged with respect to the 350 K potential temperature surface and directly compared to the $A_{y}+A_{z}$ interannual anomaly results at 350 K. To avoid confusion, we choose not to do this and, in this study, only lag the near‐surface tracer concentrations with respect to the 50 hPa pressure level in Figures 1c and 5.

## 5 Conclusions

The recent uptick in CFC‐11 near‐surface concentrations has raised questions about dynamical influences, including tropopause‐level trace gas transport and stratosphere‐troposphere exchange. The modulation and variability of this stratosphere‐troposphere exchange on interannual timescales of 1 to 5 years, over which stratospheric dynamical influences are communicated to the surface, is of particular interest.

In this study, we extend understanding of stratospheric dynamical influences on N_2_O and CFC‐11 near‐surface concentrations. Contributions from modeled advection, mixing and chemistry are distinctly resolved by using a TEM tracer budget and by analyzing its tracer advection terms on isentropic surfaces. We identify dynamical conditions under which the modeled interannual advection anomaly strengthens. Consistent with previously published results, we find that during the easterly QBO phase, the interannual advection of tracer concentrations in the extra‐tropics and at mid‐latitudes is positive in this model. Conversely, the westerly QBO phase coincides with negative interannual advection of tracer concentrations. Various physical processes influence the modeled advection in the easterly compared to the westerly phase, including the QBO‐induced secondary mean‐meridional circulation in the lower stratosphere and the modulation of the Northern Hemisphere polar vortex by the QBO; however, quantifying the contributions of these physical mechanisms is beyond the scope of this paper.

The positive stratospheric tracer advection associated with weaker downward extratropical circulation in the easterly phase of the QBO flushes air more strongly from the stratosphere into the troposphere compared to the westerly phase of the QBO. The positive interannual $A_{y}+A_{z}$ in the lower stratosphere implies a positive lower stratospheric $\partial_{t}\chi$ interannual anomaly during the easterly phase (Equation 1, Figure 1). As N_2_O and CFC‐11 are depleted in the stratosphere, the air brought down from the stratosphere has lower tracer abundances than tropospheric air, thus diluting tropospheric concentrations. Therefore, we expect stronger dilution of tropospheric concentrations in the easterly compared to the westerly phase. The interpretation based on calculations from the model output is supported by advection calculations from observations of N_2_O and CFC‐11 concentrations from ACE measurements together with reanalysis fields. Accordingly, the modulation of the near‐surface timeseries of tracer concentrations, presented as the 5‐year running mean subtracted from the 1‐year running mean, generally peaks in the westerly phase when stratosphere–troposphere advection is weakest and consequently when the least dilution of tropospheric concentration occurs. Given that these simulations employ surface trace gas boundary conditions, future work could compare results from observations and simulations forced by observed emissions.

We also find disruptions to the general pattern described above, which we relate to phase unlocking of the ≈28‐month QBO cycle from the 12‐month annual cycle. The QBO has a different phase relationship with the annual cycle and returns to its current point in the annual cycle once every ≈7 years, resulting in periods when the QBO “unlocks” from the annual cycle. We use the 300 years of simulations in our 10‐member, 30‐year ensemble with a regularly repeating QBO cycle to explore these phase relationships in different seasons and hemispheres. In our calculations from this ensemble output, we find seasons when the QBO and seasonal cycle are consistently locked, such as spring seasons in both the Northern and Southern Hemispheres for interannual advection of N_2_O and CFC‐11, and seasons when the QBO and seasonal cycle frequently unlock, such as summer and spring in the Northern Hemisphere for N_2_O interannual advection.

Tracer‐tracer differences between phase unlocking patterns of N_2_O and CFC‐11 interannual advection during Northern Hemisphere fall and winter can likely be attributed to how the model's Brewer Dobson Circulation transports each chemical species. As CFC‐11 loss occurs lower down in the stratosphere than N_2_O loss, CFC‐11 advection is hence more strongly influenced by the lower branch of the BDC while N_2_O advection is more strongly affected by the upper branch of the BDC in WACCM. In the Northern Hemisphere where the upper branch of the BDC is stronger than in the Southern Hemisphere, N_2_O advection phase unlocks more frequently than CFC‐11 advection. Additionally, N_2_O advection in the Northern Hemisphere phase unlocks more frequently than N_2_O advection in the Southern Hemisphere. This suggests an association between the strength of the upper branch of the BDC and the frequency of phase unlocking. Given this association, future studies on tracer‐tracer and hemispheric differences in interannual phase locking/unlocking patterns would be an insightful avenue to highlight differences in tracer transport between models and between data assimilation schemes in reanalysis.

Our study suggests that for 8–12 months following a westerly QBO phase, near‐surface concentrations (with anthropogenic increases removed) on interannual timescales, defined in this study as 1 to 5 years, generally increase, with associated disruptions to this general pattern. Therefore, attempts to account for imprints of dynamical influences on near‐surface concentration signals should include stratosphere‐troposphere advection modulated by the QBO and its seasonally changing phase relationship.

This work augments existing studies that use a TEM tracer budget to quantify stratosphere–troposphere exchange (Abalos et al., 2020; Minganti et al., 2020). It advances the existing body of literature about synchronizations between the annual cycle of ozone concentrations and the QBO (Gray & Dunkerton, 1990) by examining phase unlocking patterns in the lower stratospheric advection of tracers. However, difficulties arise with removing a nonlinear anthropogenic increase from CFC‐11 concentrations while also retaining information about its changing phase relationship with the QBO, a difficulty that may continue in the future as banks release CFC‐11.

Phase unlocking patterns in observations need not correspond to those in these model simulations, because the observed QBO has varying periodicity and varying lengths of each phase, but these patterns appear to display similar features. Thus, taking high‐precision, direct measurements of stratospheric and near‐surface tracer concentrations over several decades and continuing to output temperatures and velocities from reanalysis are necessary to understand the irregular phase unlocking patterns in the observed atmosphere, which themselves are a crucial component of the endeavor to understand the stratospheric dynamical influence on near‐surface tracer concentrations.

## Supporting information

Supporting Information S1

Movie S1

## Data Availability

ACE data are available through the SCISAT database (ACE/SCISAT Database, 2022). AGAGE data are available at the AGAGE Data Archive (2022). Six‐hourly JRA‐55 reanalysis data is available at NCAR/UCAR (2022). WACCM model output used in this study is available at NCAR/ACOM (2022).
