# Supplementary material for: Phase Unlocking and the Modulation of Tropopause‐Level Trace Gas Advection by the Quasibiennial Oscillation
Source: J Geophys Res Atmos. 2022 Nov 10;127(21):e2021JD036142. doi: 10.1029/2021JD036142 (PMC9788321; doi:10.1029/2021JD036142)
Supplement: Supplementary file 1 — Supporting Information S1 [file JGRD-127-e2021JD036142-s001.pdf]

Phase unlocking and the modulation of tropopause-level trace gas advection by the quasibiennial oscillation

Kasturi Shah[1], Susan Solomon[1], Douglas Kinnison[2], Qiang Fu[3], David WJ Thompson[4]
[1] Department of Earth, Atmospheric and Planetary Sciences, Massachusetts Institute of Technology, Cambridge, MA 02139, USA
[2]Atmospheric Chemistry Observations and Modeling, National Center for Atmospheric Research, Boulder, CO 80307, USA
[3] Department of Atmospheric Sciences, University of Washington, Seattle, WA 98195, USA
[4] Department of Atmospheric Science, Colorado State University, Fort Collins, CO 80523, USA

Supplemental figures and movie

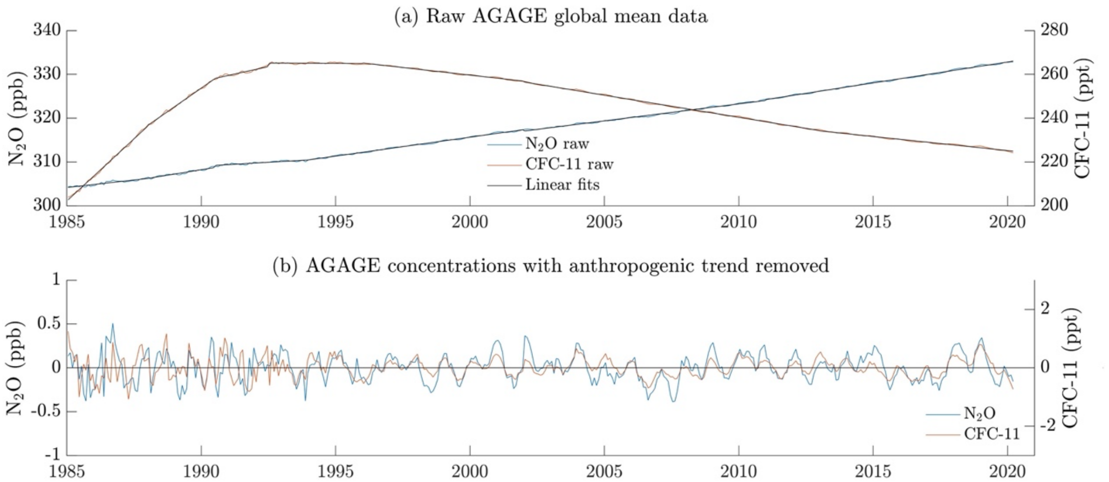

Figure S1 – Removing the long-term increases from AGAGE tracer concentrations at the surface. (a) Global mean data for N_2O (blue) overlaid by a piece-wise linear fit (black) with breakpoints in 1988, 1991, 1994, 2002 and 2012. CFC-11 (orange) is overlaid with a piecewise linear fit with breakpoints in 1989, 1991, 1993, 1995, 2002 and 2011. (b) The detrended AGAGE concentrations for N_2O and CFC-11.

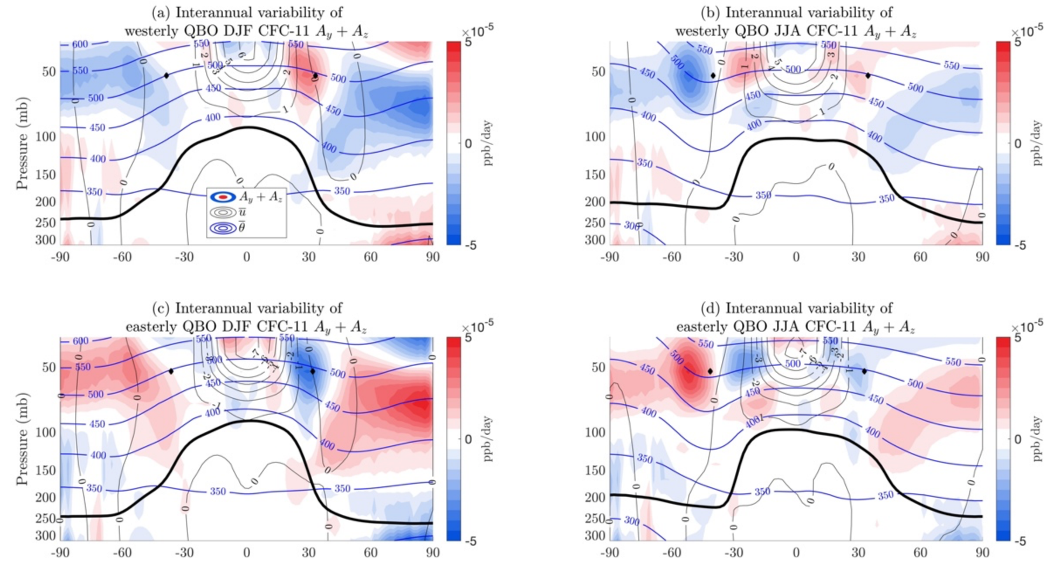

Figure S2 Same as Figure 2, but for interannual anomalies of the CFC-11 advection terms.

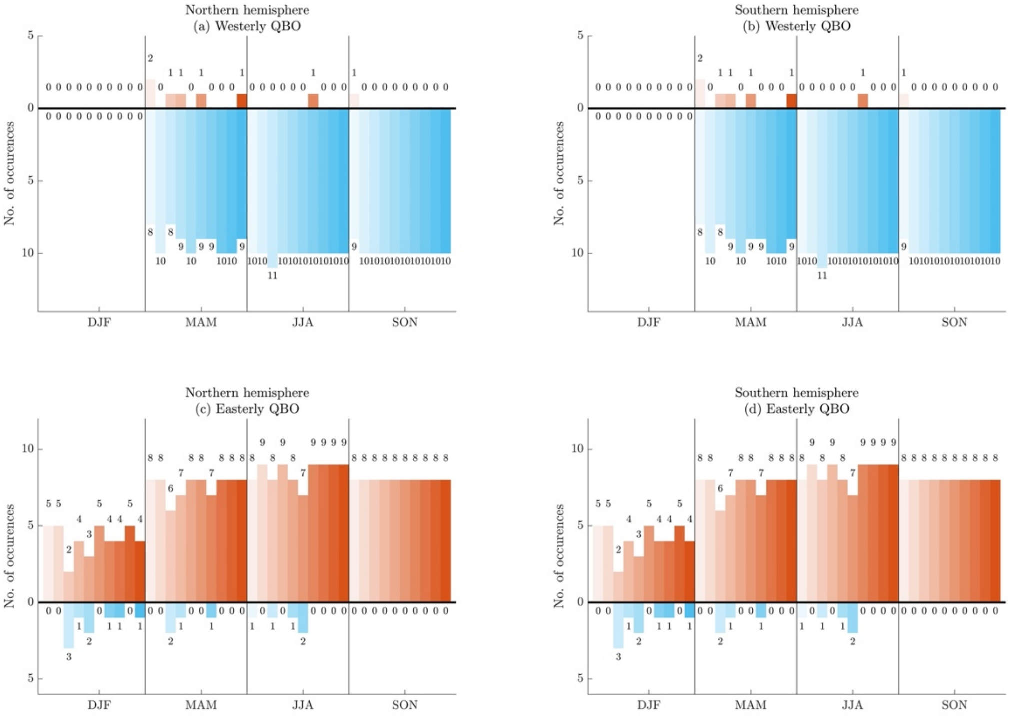

Figure S3 Same as Figure 4 but for calculations of interannual anomalies of $A_y + A_z$ of CFC-11 from WACCM.

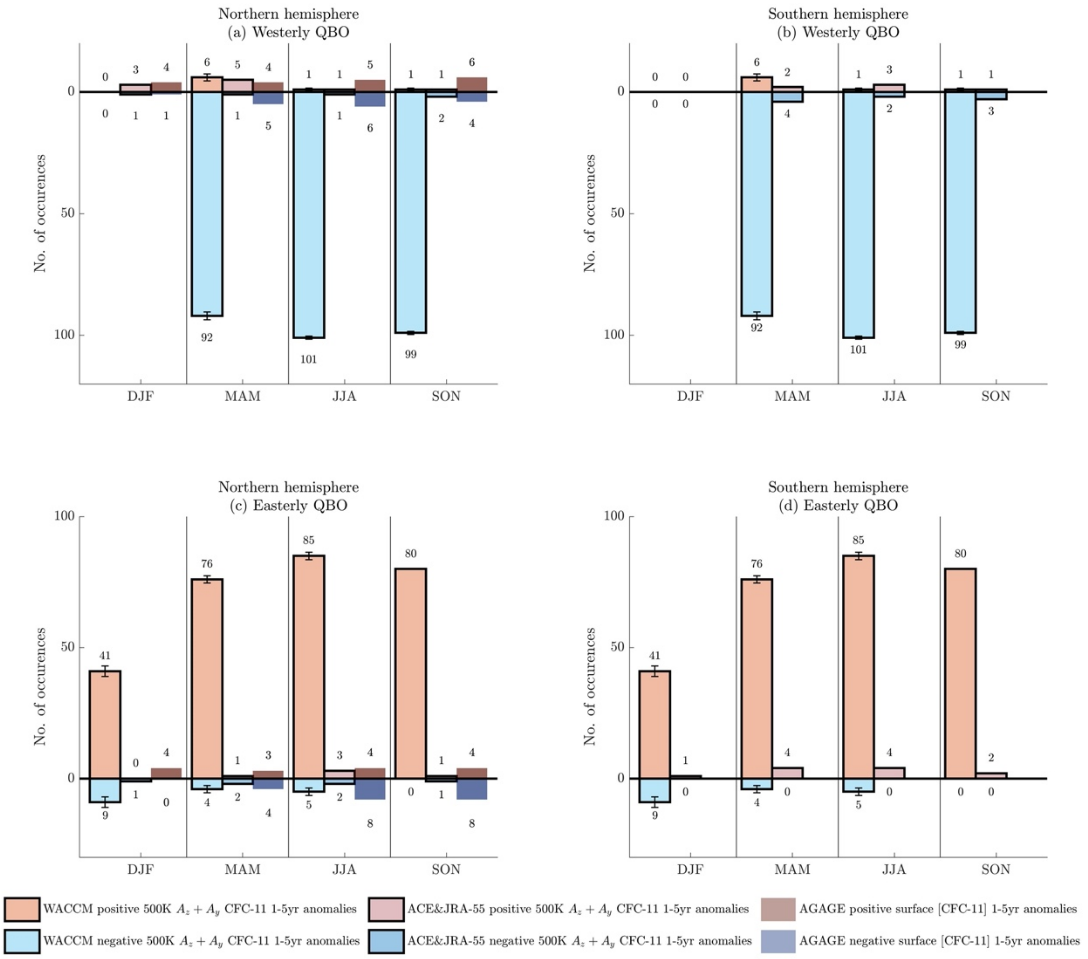

Figure S4 Same as Figure 5 but for interannual anomaly calculations of $A_y + A_z$ of CFC-11 from WACCM, for calculations of $A_y + A_z$ of CFC-11 from ACE measurements and JRA-55 reanalysis, and for CFC-11 global-mean near-surface concentrations from AGAGE. The number of total counts in each quantile for model ensemble/ACE-JRA55 results/AGAGE are: **(a)** 300/15/35 **(b)** 300/11/35 **(c)** 300/15 **(d)** 300/11.

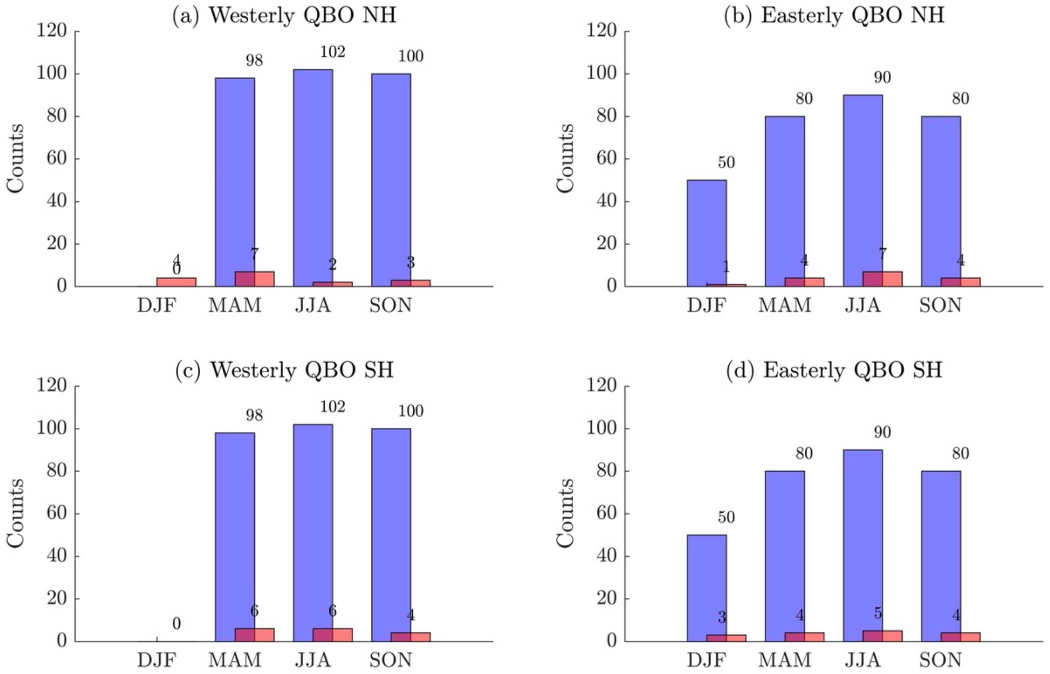

Figure S5 Seasonal partitioning of QBO phases of the WACCM ten-member ensemble (blue) and JRA55 (red). The westerly QBO is defined as the 0.75 quantile and the easterly QBO is defined as the 0.25 quantile of $\overline{u}$ at 50 hPa.

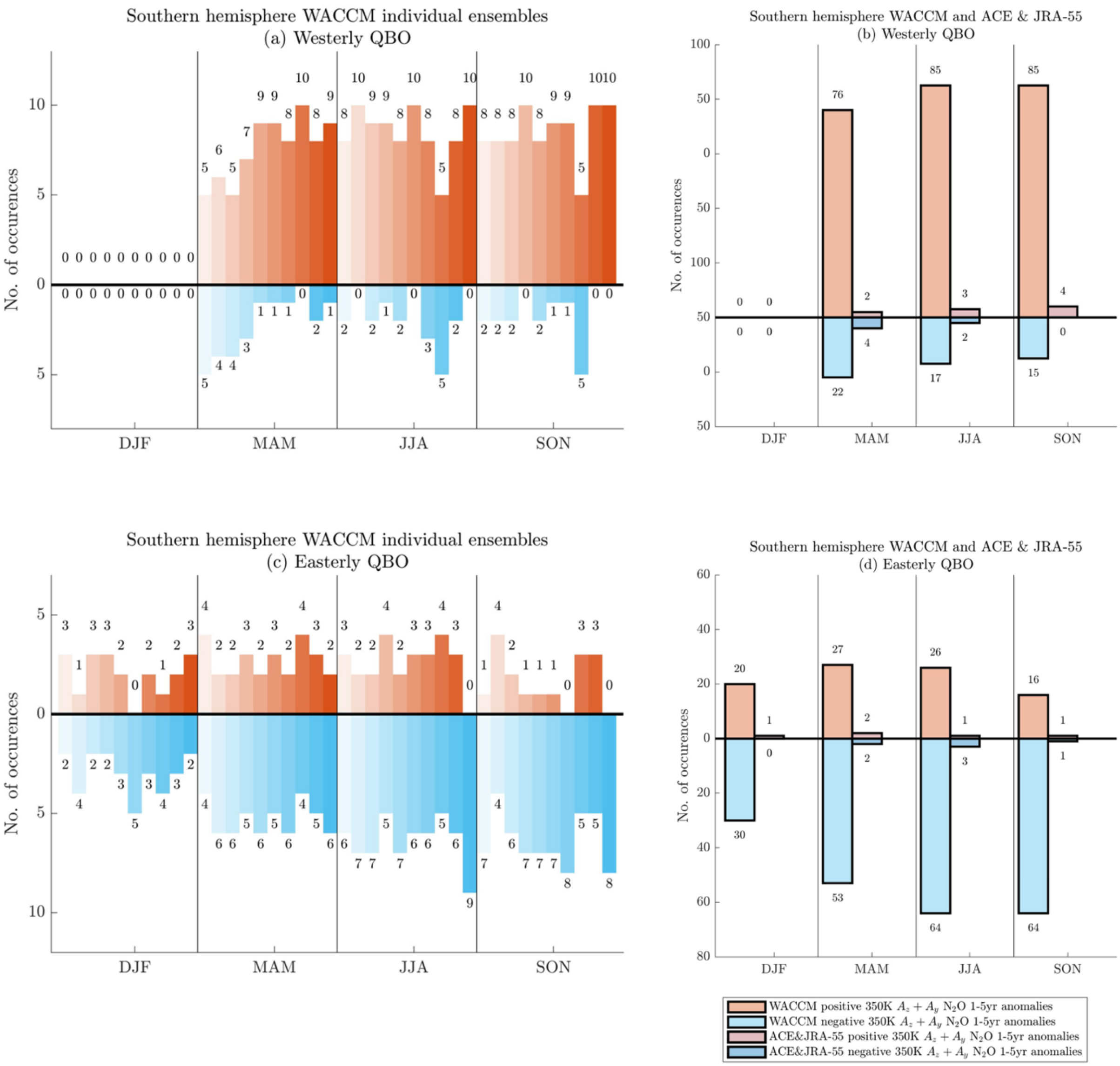

Figure S6 Same as Figure 7 but for interannual anomalies of $A_y + A_z$ for N_2O at 350K in the Southern Hemisphere.

Supplementary Movie 1

Month-by-month video of the WACCM ensemble-mean interannual anomalies of (a) zonal-mean temperature, (b) zonal-mean zonal wind, (c) zonal-mean N_2O as colour contours and $< A_y, A_z >$ as arrows, (d) zonal-mean CFC-11 as colour contours and $< A_y, A_z >$ as arrows. In each panel, the isentropes are shown as thin blue contours and the climatological tropopause as a thick black line.
